# Numerical Study of GaP Nanowires: Individual and Coupled Optical Waveguides and Resonant Phenomena

**DOI:** 10.3390/nano13010056

**Published:** 2022-12-23

**Authors:** Maria A. Anikina, Prithu Roy, Svetlana A. Kadinskaya, Alexey Kuznetsov, Valeriy M. Kondratev, Alexey D. Bolshakov

**Affiliations:** 1 Laboratory of Functional Nanomaterials, Center for Photonics and 2D Materials, Moscow Institute of Physics and Technology, 9 Institutskiy Lane, 141701 Dolgoprudny, Russia; 2 Department of Physics, ITMO University, Kronverkskii 49, 197101 Saint Petersburg, Russia

**Keywords:** nanowire, GaP, photonics, waveguide, cavity, optical coupling

## Abstract

The development of novel nanophotonic devices and circuits necessitates studies of optical phenomena in nanoscale structures. Catalyzed semiconductor nanowires are known for their unique properties including high crystallinity and silicon compatibility making them the perfect platform for optoelectronics and nanophotonics. In this work, we explore numerically optical properties of gallium phosphide nanowires governed by their dimensions and study waveguiding, coupling between the two wires and resonant field confinement to unveil nanoscale phenomena paving the way for the fabrication of the integrated optical circuits. Photonic coupling between the two adjacent nanowires is studied in detail to demonstrate good tolerance of the coupling to the distance between the two aligned wires providing losses not exceeding 30% for the gap of 100 nm. The dependence of this coupling is investigated with the wires placed nearby varying their relative position. It is found that due to the resonant properties of a nanowire acting as a Fabry–Perot cavity, two coupled wires represent an attractive system for control over the optical signal processing governed by the signal interference. We explore size-dependent plasmonic behaviors of the metallic Ga nanoparticle enabling GaP nanowire as an antenna-waveguide hybrid system. We demonstrate numerically that variation of the structure dimensions allows the nearfield tailoring. As such, we explore GaP NWs as a versatile platform for integrated photonic circuits.

## 1. Introduction

According to Moore’s law the electronic integrated circuits are reaching the dimensional fundamental limit nowadays. However, the digital data usage per capita is increasing exponentially stimulating development of the new approaches for data transfer and processing. One of the most promising methods in this field is the optical integrated circuits. Since the 1960s, laser and optical fiber technologies paved the way to optoelectronic technologies. The transition to the photonic circuitry implies an increase in the operating frequency, a decrease in sensitivity to external environmental factors, as well as a reduction in the applied power and thermal losses compared to digital electronics. However, optical technology must first prove itself to replace electronics in every respect [1]. To get to the chip scale, novel structures need to be developed. In particular, 2D [2,3], 1D [4,5], and 0D [6] structures, as well as hybrid structures based on them [7,8,9,10], are the promising candidates.

The nanostructures attract a lot of attention due to the unique properties they demonstrate both in nature [11] and fabricated in the lab [12]. The eminent part of the optical integrated circuits is based on III-V semiconductor materials due to their optical and electronic properties, which can be tailored with the proper structure geometry and chemical composition. III-V nanowires (NWs) get much attention among the variety of these structures. Vapor–liquid–solid (VLS) [13] NWs can be obtained with the use of conventional techniques such as chemical vapor deposition (CVD) [14] and molecular beam epitaxy (MBE) [15]. These methods provide very high crystalline purity and composition uniformity [16]. Moreover, it is possible to control the grown NW arrays surface density and geometry with these methods [15]. This provides a basis for the development of various elements, such as waveguides [17], nanoscale light-emitting diodes [18], cavities [4], lasers [19], up-converters [20], nanoantennas [21] and photodetectors [22].

Gallium phosphide (GaP) is a mature semiconductor material that despite indirect-gap has been successfully used in a range of solid-state light-emitting devices [23]. GaP is attractive for nanophotonic applications due to its high refractive index (*n* > 3) and transparency in the visible and infrared wavelength ranges (0.55–11 µm). GaP is characterized by large χ^(2)^ and χ^(3)^ nonlinearity, which promotes its use for the up conversion. The promising properties of GaP were demonstrated using the Kerr effect in GaP-on-insulator waveguides [24], second harmonic generation (SHG) in GaP nano-waveguides [25], orientation-patterned GaP waveguides [7], and metasurfaces [26], individual [27], and GaP NW arrays [28]. 

**Figure 1:**
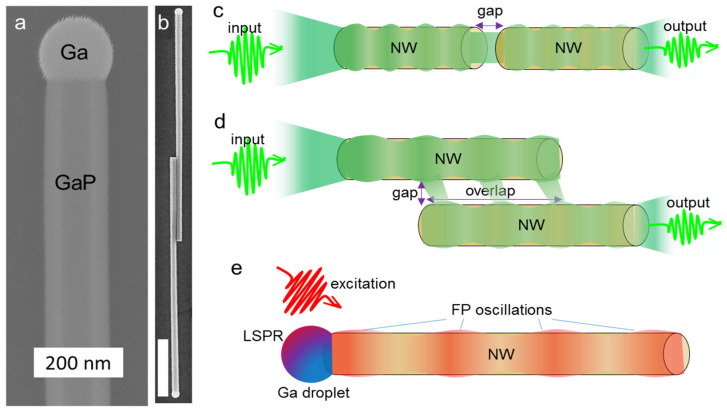
GaP NWs and phenomena understudy. SEM images of (**a**) GaP NW with Ga droplet, (**b**) planarized overlapped GaP NWs representing coupled waveguides optical system, scale bar—1 µm; schematics of the modeled systems: (**c**) two aligned GaP NWs waveguiding system, (**d**) waveguiding in parallel GaP NWs, and (**e**) size-dependent resonant phenomena in individual GaP NW.

GaP NWs represent an attractive nanophotonic platform due to the self-assembled growth nature, vast abilities to control the geometry [29] and composition [15]. Despite the indirect bandgap, GaP NWs can be diluted with As or covered with active materials such as colloidal quantum wells [30,31] to fabricate efficient nanosized light sources. These properties together with the optical phenomena provided by the Ga growth catalyst plasmonic nanoparticle (NP, see image in Figure 1a) [32] promote use of these nanostructures as both passive and active elements of the photonic circuits. Some of the intriguing possibilities including efficient waveguiding in the visible region, field confinement and light coupling were discussed previously [4,8]. For development of the photonic circuits based on GaP NWs it is necessary to study in detail peculiar phenomena related to the size-dependent effects, optical coupling between the NWs acting as waveguides (image of two GaP NWs representing the coupled waveguides is shown in Figure 1b) and plasmonic Ga nanoparticle properties. Despite the huge amount of works dedicated to the study of the GaP optical waveguides and nonlinear phenomena, quite a few works investigate the feasibility of light propagation in the self-catalyzed GaP NWs. 

The development of the integrated optical circuits based on GaP NWs necessitates detailed investigation of the near field phenomena taking place upon propagation of light through these nanostructures. Here we study numerically propagation of visible light in the system consisting of GaP NWs and demonstrate the effects of the system geometry on its optical properties. This includes size effects on the light propagation along an individual NW waveguide, optical coupling of the two aligned GaP NW waveguides dependent on the distance between the wires (see schematics in Figure 1c), and optical coupling of the NWs lying in parallel (see schematics in Figure 1d). We investigate tolerance of the GaP NWs waveguiding properties to the displacement of the elements and shed light on requirements of the fabrication processes necessary for large scale fabrication. In the end, we explore the dependence of the GaP NW and Ga NP resonant properties (Fabry–Perot (FP) and localized surface plasmon resonance, see schematics in Figure 1e) on the system geometry providing pathways for the fine-tuning of the self-catalyzed GaP NWs optical properties.

## 2. Materials and Methods

### Software

We used COMSOL Multiphysics 5.5 [33] to simulate the investigated optical phenomena. A 256Gb RAM server with Intel Core i9 CPU (Intel, Santa Clara, CA, USA) is used for simulation. The convergence plot is analyzed for each solution to remove any non-physical and erroneous solutions. The MUMPs and iterative solvers integrated in COMSOL are used. The mesh size is 1 nm (unless specified) for each design and configuration. The parametric sweep feature is used to analyze different combinations of variables. However, in some sections we use parametric sweeps to vary diameter of NWs as well as wavelengths to get more detailed analysis. We use numerical ports with TE polarization for most of our simulation. The field and power is calculated at the monitor assigned in the model. Monitor at the edge of NWs to study the power input and output and along the NW length to understand the light power distribution. The simulation is carried out in 2D geometry because of the symmetry of the NW. The perfectly matched layer (PML) is used to attenuate any reflection from the boundary of the domain. We use GaP refractive index value from [34] and for Ga from [35].

## 3. Results and Discussion

### 3.1. Peculiarities of Waveguiding in GaP Nanowires

GaP is one of the prominent semiconductor materials with large indirect bandgap and refractive index. This optical property makes it promising especially as a waveguide material that provides high field confinement and low optical losses. As discussed earlier, semiconductor NWs due to their self-assembled nature can be used as optical waveguides in the integrated photonic circuits. 

**Figure 2:**
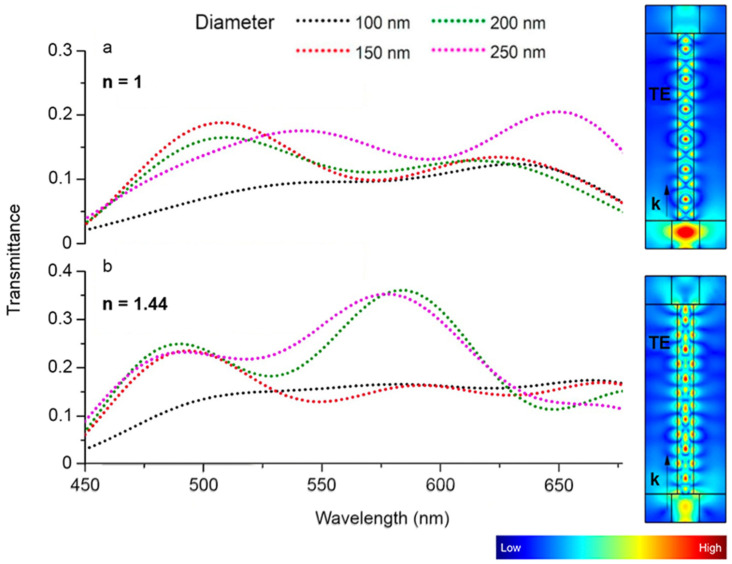
Transmission spectra of 2 μm long GaP NW having diameter of 100–250 nm in different media: (**a**) air and (**b**) refractive index of *n* = 1.44. Inset demonstrates normalized electric field distribution in 200 nm diameter NW excited by λ = 532 nm.

In the first part of our numerical study, we explore the waveguiding spectral features of individual GaP NWs having different diameters. The NW is simulated as a 2 μm long GaP cylinder surrounded by air. To study the waveguiding, we put a source port on the left side of the NW and the output port to the right of the NW (see schematics in inset, Figure 2a). Throughout the visible region (450–700 nm) we analyze transmission efficiency of the NWs having diameters of 100–250 nm as the ratio between the output power and the input power. The obtained transmission spectra are plotted in Figure 2a.

The obtained spectra demonstrate two main features regarding the NW geometry. First is the anticipated overall tendency of the transmission to increase with an increase in the NW diameter; the light is more confined inside the NW waveguide in the thicker wires. The second feature is the non-monotonous multi-peak behavior of the transmission spectra for the NWs exceeding 100 nm. This effect is the manifestation of the NW optical resonant geometry: when the NW is thick enough to guide the light inside, this light is partially reflected on both edges of the NW which leads to its interference and occurrence of the FP-like oscillations. Here we note that for the thick enough wires these modes are not pure FP due to the reflection from the NW sidewalls. This phenomenon has been reported previously with investigation of the properties of the NW-based light emitters [4]. According to the general theory of the FP cavities, these spectral modulations are inversely proportional to the cavity length and so can be finely tuned by the NW length. This conclusion is in agreement with the modeling results demonstrating that the spectral position of the transmission resonances is not affected sufficiently by the change in the NW diameter and then should be a function of the NW length. The transmission suffers an abrupt fall near 450 nm due to the rise in absorption in GaP. The overall transmission efficiency in air is maximized at 18% for λ = 500 nm and NW diameter of 150 nm and at 20% for λ = 650 nm and NW diameter of 250 nm.

The waveguiding can be enhanced with encapsulation of the waveguide in higher refractive index material. Increase in the surrounding medium refractive index provides decrease of the optical gradient at the NW edges followed by the enhanced both light coupling and outcoupling. A typical example of the cladding layer material is polydimethylsiloxane (PDMS) and silica having the refractive index (*n* = 1.44) very close to water (*n* = 1.33). The results of the modeling are presented in Figure 2b. The plot demonstrates resonant features similar to the model in air surroundings. The use of more optically dense surroundings enhances the transmission with the peak value of 35% for λ = 580 nm and NW diameter of 200 nm and 250 nm. 

Here we note that increase in the cladding refractive index increases the critical angle required for total internal reflection as the main criterion for the efficient waveguiding. So, for the thicker wires with the leading modes of high orders, this effect may lead to increase in the transmission efficiency compared to the case of the air surroundings, while for the rather thin NWs under study, this criterion is not the governing factor. In our case, presence of a more optically dense medium at the entry face of the NW promotes decrease in the reflection and consecutively increase in the transmission function of the wire waveguide. So, control over the cladding layer (refractive index) is an efficient way for the fine tuning of the NW waveguiding properties. 

### 3.2. Optical Coupling between the NW Waveguides

To develop practical photonic integrated circuits, it is important to study propagation of the optical signal between the photonic elements. As GaP NW is assumed as an element for fabrication of both emitters and waveguides, it is of interest to study the optical coupling between two adjacent NWs. The coupling of the NWs in an optical chip can be considered as a counter-part of impedance matching in electronics promoting efficient transfer of power between the elements. 

**Figure 3:**
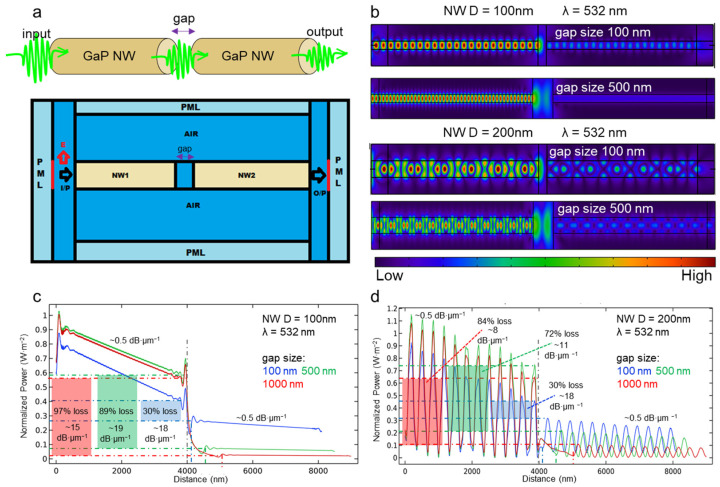
Simulation of the light propagation through two aligned GaP NW waveguides: (**a**) system schematics and geometry of the simulation, mesh size 1nm, (**b**) field patterns (normalized electric field distribution) for the system configurations with 100 nm and 200 nm thick master-slave NWs, 100 nm and 500 nm gap between them, (**c**) light power distribution along the 100 nm thick NWs for three different gaps, and (**d**) light power distribution along the 200 nm thick NWs for three different gaps (Input power = 1 W/m^2^).

In the first part of this study we simulate coupling efficiency by changing the gap between two NWs placed along one axis (see schematics in Figure 3a) with the source located at the left edge of the input NW and the output port at the right edge of the output NW. There are two major states of the NW optical property regarding our study which depend on its cross-section: waveguiding (with the field confined inside the NW) and non-waveguiding. In this work, we consider two different diameters of the adjacent NW waveguides: 100 nm (close to the cut-off) and 200 nm (well above the cut-off) under 532 nm illumination. The length of NWs in the model is 4 μm.

As a reference, propagation of 532 nm light in air was simulated. For this purpose, we took the same model displacing the GaP NWs with air. The modeling results demonstrate spatial attenuation of the signal power at the level of 8 dB·μm^−1^. In the next step we simulate the propagation of light through two 100 nm thick GaP NWs. The calculated light power distributions along the system axis for three values of the gap between the NWs—100 nm, 500 nm and 1000 nm are presented in Figure 3c. This plot demonstrates efficient light transmission through the NW providing sufficiently lower losses at the level of 0.5 dB·μm^−1^ (indicated in Figure 3c,d). When the light scatters at the right edge of the input wire, the power starts to attenuate fast with the losses of ~15–19 dB·μm^−1^ depending on the gap between the NWs. Interestingly, these values exceed the estimated losses in air by two-fold. Such a behavior is the result of the interference of the light reflected from the NWs edges in the gap. As such, the transmission function of the two coupled wires should be tailored in order to minimize the destructive interference.

In the gap, the power decreases by 30% for the smallest considered gap of 100 nm. The losses in the gap increase up to 97% when the gap reaches 1 μm. For the visualization of the coupling effect we plot the field distribution maps along the NWs for two different gaps of 100 nm and 500 nm. The plot demonstrates oscillations of the field related to the FP oscillations provided by the resonant geometry of the NWs. Field pattern of the master NW also changes with the gap variation, due to reflection in between the NWs.

In the case of 200 nm thick NWs (Figure 3d), strong oscillations of the propagating light power are observed along the axis for all of the considered gaps. To get into details of the observed phenomenon we plot the field pattern along the wires depicted in Figure 3b. The plot demonstrates a more complex resonant pattern compared to the thinner wire which relates to the reflection of light from the NW sidewalls leading to the dips in the spatial power distribution in Figure 3d. 

In order to quantify the light transmission between the coupled NW waveguides we compare maximum field power at the adjacent NWs edges. This comparison gives 30% signal losses for the 100 nm gap which is similar to the previous case of the thinner waveguides. Interestingly, the losses increase slower with the gap compared to the first case with the maximum losses of 84% for the 1000 nm gap. We assume this is due to the more efficient coupling of the transmitted signal into the 2nd NW related to the increase in the NW cross section.

The obtained data demonstrate that in order to provide the efficient coupling between the two GaP NW waveguides, the distance between them should not exceed 100 nm, which is assumed technologically feasible value as there are several nano-manipulation techniques like AFM, optical tweezer and electron beam lithography providing demanded precision of the manipulation [36]. This result shows good tolerance of the optical system based on two aligned coupled GaP NWs to their axial displacement. Reduction of the gap size to some extent should enhance the coupling, however the light reflection in the gap is the limiting factor. Use of the tapered NWs can probably solve the problem, which will be the future prospect of this task.

### 3.3. Effects of the Nanowires Relative Position on the Waveguiding

**Figure 4:**
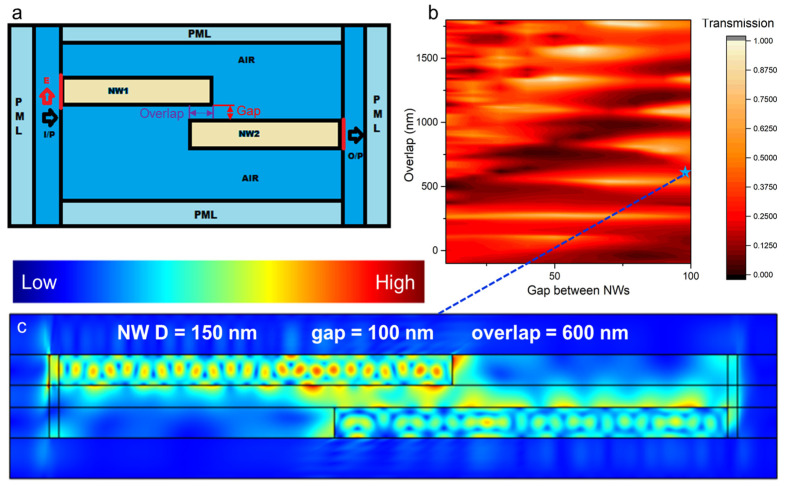
Simulation of the light propagation through displaced GaP NWs: (**a**) model geometry, (**b**) colormap showing normalized transmission for different gap and lateral overlap, and (**c**) normalized E-field distribution plot showing coupling of two 2 μm NWs separated by 100 nm gap and overlapped by 600 nm.

In the previous section, we examined the effect of the gap on the coupling of two aligned NWs. In this part, we investigate tolerance of the two NWs coupling to the mixed displacement which is a more realistic situation. A model schematic is presented in Figure 4a. Here two 2 μm long and 150 nm thick NWs are positioned in parallel with their axes at a distance referred hereafter as gap and exhibiting overlap. 

The idea in this section is to understand the cross talk or coupling between the two parallel waveguides. Similar to electronic circuits, it is worth studying how close we can put the two parallel optical waveguides without them affecting the field inside each other. On the other hand, if we want to develop a feedback loop, we need to know how close we should keep the NWs laterally for better coupling. In all of the cases it is necessary to choose an above cut-off wire providing efficient field confinement and negligible leakage losses.

In the modeling, as previously, we use input and output sources, the first one at the left edge of the input NW and the second at the right edge of the output NW. To study the effect of both considered geometrical parameters on the efficiency of the light (532 nm) transmission in the system, we calculated the transmission efficiency as a ratio of delivered and radiated power varying the gap and the overlap. As a result, the efficiency map shown in Figure 4b was plotted. The plot demonstrates that the coupling efficiency is a non-monotonous function of both geometric parameters. We discuss this effect hereafter.

First, let us consider the zero gap shear of the map. Here, the maximum transmission efficiency is found at only 30% with a very fast decay of the transmission efficiency with increase of the gap. As such, the loss of the NWs alignment along one axis leads to the very fast attenuation of the transmission. On the other hand, this misalignment can be efficiently compensated by the increase in the NWs overlap. As can be seen, an overlap of 260 ± 15 nm brings stable efficient transmission with the gap size up to 90 nm. Further increase of the overlap up to 370 nm leads to the drastic fall of the transmission efficiency. The efficiency dependence on the gap becomes more complex with increase of the overlap demonstrating series of the transmission peculiarities including both bright spots (efficient transmission) and dark spots (loss of transmission).

To get insight on these peculiarities we plot the field pattern of the light propagating through the studied system with 100 nm gap and 600 nm overlap (Figure 4c). Here, complex field distribution due to the interference governed by the multiple reflection interfaces is depicted. This includes F-P-like oscillations inside the NWs and hotspots observed in the overlap region. Thus, we conclude that due to the cavity action of the NWs, constructive (high transmission mode) and destructive (low transmission mode) interference can be observed. This complex phenomenon, depending on the dimensions of the NWs, their relative position and the radiation wavelength, opens intriguing possibilities for the control over the transfer function of the system. This includes fabrication of the nanoscale optical filters with an ability to mechanically control the spectral properties of the device, efficient optical measuring equipment based on the strong effect of the system geometry on its transmittance and brings a roadmap for the fabrication of the novel nanophotonic waveguides.

### 3.4. Size-Dependent Resonant Effects

Plasmonic nanoparticles (NPs) exhibit a peculiar electromagnetic response which can be tailored with the change in the size of the NP. However, it is cumbersome to fabricate metal-dielectric hybrid systems with the multistep lithography or nano-manipulation techniques. VLS-grown NW is a self-assembled hybrid plasmonic-semiconductor nanostructure with conjugated metallic sphere and cylindrical NW. In our previous works we discussed possible effects of the plasmonic NP resting on the top edge of the NW on the light propagation and field confinement [8]. Here we explore numerically how the NW and Ga catalyst NP dimensions affect the optical phenomena at the nanoscale.

There is a linear ratio between thickness of the NW and diameter of a catalyst NP which depends on the droplet contact angle which is, in turn, governed by the relation between the surface energies of the NW crystal lattice and the NP [37,38]. Typical contact angle of the catalyst NP exceeds 120°, so in the calculations we used the ratio between the NP/NW diameter at the level of 1.25. For detailed analysis we considered 4 different thicknesses of the NW, namely, 80, 120, 160 and 200 nm, corresponding to the most represented values allowed by the epitaxial growth techniques. 

**Figure 5:**
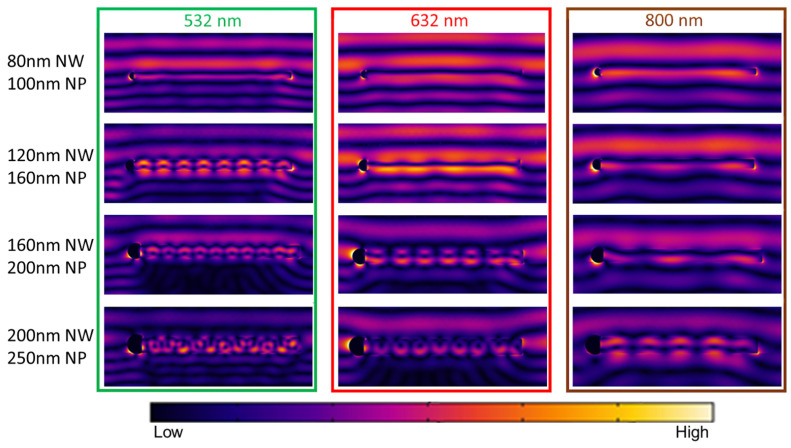
Investigation of the size-dependent resonant optical phenomena in GaP NW with Ga NP (circular segment on the left edge of the wire). The plots demonstrate normalized (E/E_0_) near field distribution of 532 nm, 632 nm and 800 nm plane waves scattered on the NWs of different diameters.

In this study we consider the near field distribution provided by the scattering of a plane wave on an individual NW. To make the analysis straightforward, we took three different wavelengths in the visible and near IR; the most widely presented in the modern optical setups are 532 nm, 632 nm, and 800 nm. The results of the modeling are presented in Figure 5. The images demonstrate some tendencies of the scattering pattern provided by the change in the nanostructure dimensions.

First, let us consider 532 nm illumination. A total of 80 nm thick NW exhibits only a weak scattering of the plane wave which is also clearly seen for the other two illumination wavelengths. Increase of the NW diameter up to 120 nm brings strongly expressed field oscillations along the NW axis. As previously, this effect corresponds to the occurrence of the mode inside the NW, rather than along its surface, followed by the cavity action of the NW and manifestation of the F-P-like oscillations. This phenomenon is observed for the other considered wavelengths with the thicker wires as anticipated (λ = 632 nm for 160 nm thick NW and λ = 800 nm for 200 nm NW). Further increase of the NW diameter up to 160 nm does not sufficiently change the field pattern - the oscillations remain, while the scattering efficiency increases which is clearly seen by the occurrence of the dark pattern below the wire. This phenomenon is also observed with 200 nm thick NW and 632 nm excitation. With λ = 532 nm illumination drastic change of the field pattern is documented in 200 nm thick wire which is a manifestation of the more complex cavity action of a larger wire promoting reflection both at the edges of the NW and its sidewalls. As to the plasmonic phenomena, the effect of the NP size on the plasmonic behavior is rather interesting. For 532 nm excitation, only a weak localized plasmon is observed with 200 nm Ga NP. This resonance does not coincide with the occurrence of the FP resonances corresponding to 120 nm thick NW. The most prominent LSPR is found for 200–250 nm Ga NP. Noteworthy, occurrence of the LSPR in 160 nm thick NW is accompanied with the FP oscillations which makes this resonant behavior more prominent. For the largest λ = 800 nm, surprisingly, the LSPR is found prominent even with small 100 nm Ga NP and vanishes in 200 nm thick NW, while F-P oscillations become prominent. 

From the results obtained, it can be concluded that for an arbitrary wavelength there is a certain NW diameter that promotes efficient field confinement on the NW surface or the wave scattering. In addition, with an increase in the NW thickness, resonant modes appear with the formation of resonant hot spots. Together with the FP resonance, the plasmonic response arises. Depending on the excitation wavelength and NW geometry, this response can either be excited together with FP resonances or not. Thus, it is possible to trigger these resonant effects together and separately.

## 4. Conclusions

To conclude, we explore numerically size-dependent optical phenomena in GaP NWs. In the first part of our study, we demonstrate that the GaP NW waveguiding efficiency is the function of the NW dimensions. As NW acts not only as a waveguide, but also exhibits cavity properties, the transmission function of the structure possesses spectral features. We then study efficiency of the optical signal transfer between the two adjacent NWs to demonstrate high transmission efficiency for the aligned NWs at a distance smaller than 100 nm. The optical coupling between the two GaP NWs is then studied in terms of the more complex relative position. In this situation, a strong effect of the interference provided by the multiple reflections from the edges and sidewalls of the NWs on the transmission efficiency is observed. Due to this, variation of the NWs relative position provides the transmission function peculiarities including high and low transmission states. In the end, we explore resonant phenomena studying the near field distribution of the plane wave incident on the NW and demonstrate interesting effects related to the size dependent plasmonic and cavity resonances.

According to the explored phenomena, the self-assembled metal-semiconductor Ga-GaP NWs represent the unique and versatile platform for the photonic chips providing fine tuning of the waveguiding properties, filtering behavior and field confinement via control over the system geometry. 

## Data Availability

Not applicable.
